# The Potential Impact of Neuroimaging and Translational Research on the Clinical Management of Lacunar Stroke

**DOI:** 10.3390/ijms23031497

**Published:** 2022-01-28

**Authors:** Salvatore Rudilosso, Alejandro Rodríguez-Vázquez, Xabier Urra, Adrià Arboix

**Affiliations:** 1 Comprehensive Stroke Center, Hospital Clínic of Barcelona, 08036 Barcelona, Spain; srudilos@clinic.cat (S.R.); alrodriguez@clinic.cat (A.R.-V.); xurra@clinic.cat (X.U.); 2 Cerebrovascular Division, Department of Neurology, Hospital Universitari del Sagrat Cor, Universitat de Barcelona, 08034 Barcelona, Spain

**Keywords:** cerebrovascular disease, stroke, ischemic stroke, lacunar stroke, small vessel disease, recent small subcortical infarcts

## Abstract

Lacunar infarcts represent one of the most frequent subtypes of ischemic strokes and may represent the first recognizable manifestation of a progressive disease of the small perforating arteries, capillaries, and venules of the brain, defined as cerebral small vessel disease. The pathophysiological mechanisms leading to a perforating artery occlusion are multiple and still not completely defined, due to spatial resolution issues in neuroimaging, sparsity of pathological studies, and lack of valid experimental models. Recent advances in the endovascular treatment of large vessel occlusion may have diverted attention from the management of patients with small vessel occlusions, often excluded from clinical trials of acute therapy and secondary prevention. However, patients with a lacunar stroke benefit from early diagnosis, reperfusion therapy, and secondary prevention measures. In addition, there are new developments in the knowledge of this entity that suggest potential benefits of thrombolysis in an extended time window in selected patients, as well as novel therapeutic approaches targeting different pathophysiological mechanisms involved in small vessel disease. This review offers a comprehensive update in lacunar stroke pathophysiology and clinical perspective for managing lacunar strokes, in light of the latest insights from imaging and translational studies.

## 1. Introduction: Clinical Relevance and Aims of the Review

Lacunar ischemic strokes are caused by small infarctions that occur in regions supplied by one perforating artery and represent from 11 to 27% of acute strokes, according to different series [1]. Lacunar strokes have milder symptoms than strokes, due to large vessel disease, and mortality is exceptional during hospitalization. However, about 20% of patients who had a lacunar stroke will present a recurrent cerebrovascular event, 25% will not survive, and 30% will have some degree of functional dependence at five-year follow-up [2]. About half of the patients with a first-ever lacunar ischemic stroke have mild cognitive impairment of subcortical vascular features, and its presence may be a predictor of subcortical vascular dementia in the medium-long-term [3]. Lacunar strokes are not isolated cerebrovascular events, but often represent the tip of the iceberg of a systemic disease affecting the microcirculation, defined as small vessel disease (SVD), which is considered to be the second cause of dementia, as well as the cause of other severe neuropsychiatric disorders, extrapyramidal symptoms, and frailty in the elderly [4]. Despite the high prevalence of lacunar strokes, and the socio-economic impact related to serious long-term-prognostic implications, no specific SVD treatment is available and most of the treatments do not differ from the management of non-cardioembolic ischemic strokes. Notwithstanding, the development of new imaging techniques, or refinement of existing ones, is providing fruitful insights into the diagnosis and pathophysiology of lacunar strokes [5]. Modeling SVD mechanisms and reproducing lacunar strokes in animal models is challenging [6,7], but translational research remains crucial for identifying new therapeutic targets and developing potential therapeutic agents. The review aims to provide a clinical approach to lacunar ischemic stroke management and future direction from imaging and translational evidence. Hence, we addressed how neuroimaging may assist clinicians in diagnosing and managing patients presenting with a lacunar stroke and reviewed the value of new biomarkers and insight from translational studies.

## 2. Terminology and Correlations between Histopathological, Clinical and Imaging Definitions

**Figure 1:**
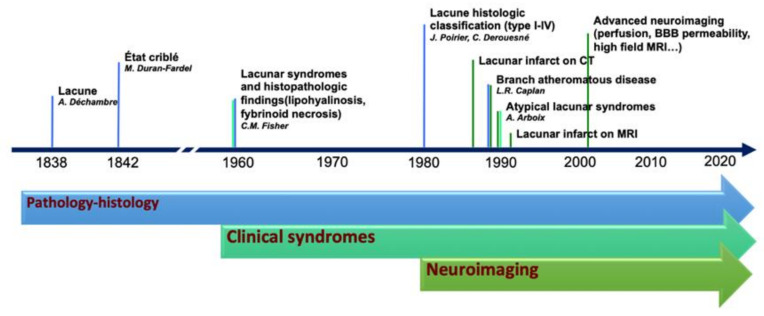
Historical evolution of the knowledge in lacunar strokes.

The terminology adopted to describe small cerebral infarcts, in the territory of perforating arteries, counts tens of different terms that have been used in research and clinical practice [5]. The discrepancies in the terminology and classification of SVD markers are, in part, the result of the integration of terms deriving from anatomical, histopathological, clinical, and radiological fields, which evolved from the first anatomopathological observation, at the end of the 19th century, to the latest neuroimaging techniques capable of assessing single perforating artery morphology and function (Figure 1). 

**Figure 2:**
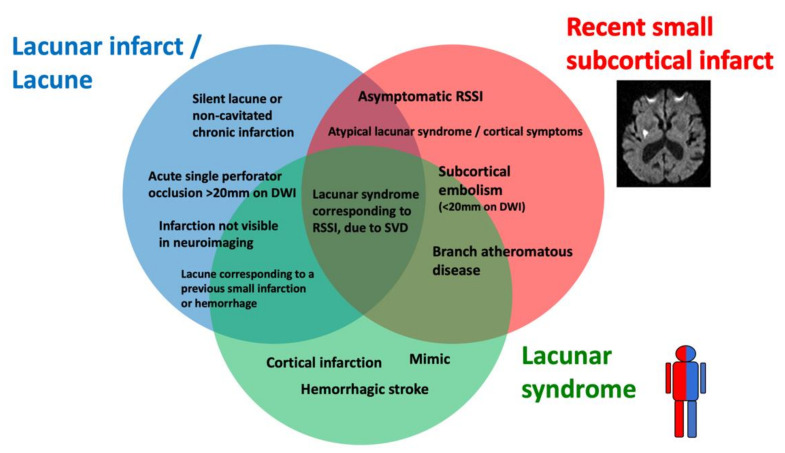
Histological–clinical–radiological correlations in lacunar strokes.

Lacunar ischemic stroke is a term used to define an acute neurological focal deficit, typically a lacunar syndrome, due to ischemia occurring in a small brain region (<15 mm) supplied by a single perforating artery, consistently with the lacunar hypothesis of an intrinsic small arteriolar disease [8]. From an etiopathological perspective, a lacunar infarct may appear as a small, incomplete infarction in different stages, from early parenchymal rarefaction and variable inflammatory cells and reactive gliosis (type 2a) to complete neuronal loss, spongiosis, and, finally, complete cavitation (type 1a) [9]. The perforating arteries may show typical arteriosclerotic concentric media thickening of the branches smaller than 200 μm, lipohyalinosis, while the more proximal branches (up to 800 μm) may show microatheromas and microvascular thrombosis [8,10]. On neuroimaging, the lacunar infarcts may correspond to small subcortical lesions with an ischemic appearance, on either CT or MRI, while the small perforating arteries are not visible using conventional imaging acquisitions. It is generally assumed that lacunar syndromes represent the clinical manifestation of a perforating artery’s occlusion, due to progressive lipohyalinosis and superposed microthrombosis, while neuroimaging provides an in vivo surrogate of the small infarction in the brain. Nevertheless, the correlation between clinical syndromes, as well as radiological and histological findings, is not absolute (Figure 2). For example, the identification, on imaging, of an acute cortical stroke necessarily excludes the SVD etiology of the stroke, even if the patient presented with a classical lacunar syndrome. On the other hand, a small subcortical infarct on imaging might be caused by mechanisms other than arteriosclerotic SVD, such as an embolism or large vessel atherosclerotic plaques occluding perforating artery branches. Lacunar infarcts and lacunes may be clinically silent or misrecognized, while disabling lacunar syndromes may be produced by infarcts so small that they might be missed, even when using techniques with high spatial definition, such as an MRI. Histopathological findings include small infarctions in the brain parenchyma, associated with typical lesions of the perforating vessels, such as lipohyalinosis, arteriolar disorganization, and perivascular edema [8,11]. However, many years might separate the stroke event from the post-mortem study, and clinical–histological correlations might be challenging to establish. Lacunes are small cavities filled by cerebrospinal fluid (CSF) in the subcortical white matter or gray matter structures that might represent the final ischemic stage after a perforating artery occlusion but are also difficult to differentiate from enlarged perivascular spaces in imaging studies. 

**Figure 3:**
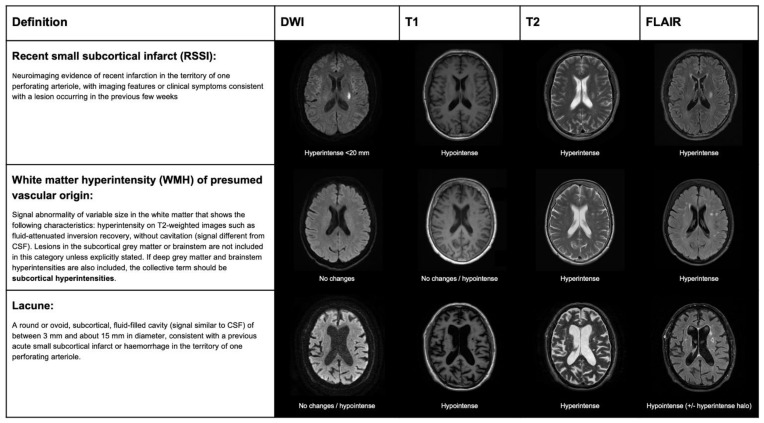
STandards for ReportIng Vascular changes on nEuroimaging (STRIVE) classification for ischemic lesions on MRI, produced by lacunar stroke.

The terminology and definitions used to classify different types of strokes, as well as the different features of SVD, need to be homogenous in research to simplify systematic search strategies and improve the external validity of observational studies and randomized clinical trials (RCTs). Several clinical classifications exist, based on clinical and radiological criteria, to differentiate lacunar strokes from strokes with other etiology. The etiology of stroke subtype may be defined according to different classifications that are based on clinical and radiological criteria. In the Trial of Org 10172 in Acute Stroke Treatment (TOAST) [12], a stroke due to small artery occlusion (lacunar) should present with a traditional lacunar syndrome and have no lesion on imaging, or a subcortical ischemic lesion smaller than 15 mm, in the absence of a mayor cardioembolic source or arterial stenosis >50% on vascular imaging. The atherosclerosis, SVD, cardiac pathology, other causes, dissection classification (ASCOD) [13], and causative classification system for acute ischemic stroke classification (CCS) [14] define the grades of likelihood for SVD etiology. According to the ASCOD definition, the probability of lacunar stroke etiology depends on the presence of a compatible lesion on imaging and other radiological signatures of SVD, such as lacunes or white matter lesions. According to the CCS classification, the probability of lacunar stroke etiology (evident, probable, or possible) is based on the evidence of a consistent lesion on imaging and lacunar syndrome presentation in the absence of alternative causative mechanisms. The standards for reporting vascular changes of neuroimaging (STRIVE) classification was proposed, in order to standardize the different features of SVD in neuroimaging, rather than differentiating lacunar strokes from other strokes subtypes. According to this classification, mostly based on MRI, the result of an occlusion of one perforating artery is classifiable as recent small subcortical infarct (RSSI), white matter hyperintensity (WMH), or lacune, according to clinical–radiological criteria (Figure 3). 

The histopathological features of lacunar infarcts were described by Fisher [8], and a classification for the different appearance of these infarcts, depending on the stage or presence of hemorrhagic features, was proposed by Derouesné and Poirer [9]. However, there is still no consensus for the terminology and reporting of SVD features in histology and their correspondence with imaging features [11,15].

In this review, we used the term RSSI to refer to neuroimaging evidence, according to the STRIVE definition. We also maintained the term lacunar stroke or syndrome, as appropriated, exclusively to refer to clinical events (i.e., acute management context). In contrast, lacunar infarction (non-cavitated lesion, either acute or chronic) and lacune (chronic cavitated lesion <15 mm) were preferred for pathological findings or assumptions.

## 3. Mechanisms of Lacunar Strokes: From Pathology Studies to Advanced Neuroimaging

Lacunar ischemic strokes are highly associated with hypertensive arteriosclerosis and other vascular risk factors [16,17,18]. However, about 15–30% of patients with lacunar ischemic strokes had no history of hypertension, suggesting that other vascular risk factors, including aging, and complex mechanisms affecting the microvascular function might play a significant role in the pathogenesis of lacunar stroke. Some of these potential mechanisms that may contribute to perforating artery occlusion, and how they might represent a possible target for therapeutic interventions, are summarized in Table 1. 

Lacunar infarcts are one of the distinctive markers of SVD, including both sporadic [16] and monogenic SVD types (such as cerebral autosomal dominant arteriopathy with subcortical infarcts and leukoencephalopathy (CADASIL) [48,49,50]) and other rare hereditary diseases [51]. In histopathological studies of lacunar infarcts, the perforating arteries typically show thickening of the media, lipohyalinosis, segmental arterial disorganization, and fibrinoid degeneration [18]. However, the histopathological findings represent the end stages of the disease, and the correlations with the clinical event are difficult to establish in most cases. For example, in the pathology studies conducted by Fisher in 114 patients with lacunar infarcts, the perforating arteries supplying the lacunar infarct/lacune had no vascular occlusion, in only 10% of the cases, and only 4 patients had no history of hypertension [8]. A recent systematic review of 39 pathology studies, including more than 4000 lacunar infarcts, highlighted that occlusion of the supplying perforating arteries was found only in a minority of cases, and the prevalence of hypertension was lower, although still higher than 50% of patients [11]. This discordancy could result from therapeutic advances in stroke prevention, such as hypertensive treatment, which could reduce the likelihood of presenting severe progressive stenosis of perforating arteries, while other alternative mechanisms, including embolic occlusions, might have prevailed. However, these mechanistic interpretations are drawn from evidence obtained in post-mortem studies conducted, in most cases months or years after the clinic event, when many factors, including remodeling of the microvasculature, changes in blood flow, and antithrombotic therapies, may have altered the structural features of the small vessels. 

Neuroimaging of lacunar stroke has the main advantage of shortening the time from symptoms onset to examination, providing imaging with high temporospatial accuracy and offering insights on the mechanisms occurring during infarction and on the course over time in longitudinal studies. However, imaging evidence has to be considered indirect in most cases, especially in SVD studies, as the small branches of perforating vessel are not visible, not even using high-definition MRI (although the main branches may be visualized using high resolution MRI angiography at 7T [27]). Much information provided by imaging studies is difficult to generalize and depends on many factors, including different criteria to define markers in imaging, acquisition protocols, processing software, and interobserver reliability. Therefore, new markers in imaging research studies need to be tested through various steps of validation. In brief, a newly discovered marker needs a proof of concept (does the marker measure a specific change related to a disease process?) and proof of principle (discriminates cases vs. controls, severity, or prognosis). The technique to assess the marker should be repeatable (precision under the same operating conditions) and reproducible (precision under different operating conditions). Finally, the marker should be effective as an endpoint for clinical studies (i.e., surrogate of a clinical endpoint) and cost-effective for use in large multicentre clinical trials. However, only a few markers in imaging used in SVD research meet most of these validation criteria. More detailed information on the validation of imaging markers in SVD is available in the harmonizing brain imaging methods for vascular contributions to neurodegeneration (HARNESS) position paper [52]. 

Deep perforating arteries are currently considered end-terminal vascular territories that supply subcortical white matter and deep grey structures. According to this classical theory, the occlusion of a perforating artery would irreversibly lead to infarction of the whole tissue, supplied by an occluded perforating artery within a few minutes after occlusion [53]. However, about 20% of patients with a lacunar stroke presented a transient ischemic attack in the previous hours or days [54], and patients presenting with a lacunar syndrome may recover without showing any ischemic lesion on brain imaging [55]. Perfusion studies on CT and MRI showed that small areas of hypoperfusion (but not a complete absence of it) are visible in some patients with a confirmed RSSI on follow-up imaging, including areas of potentially viable tissue (ischemic penumbra) [43,44], in contrast with the hypothesis of a complete flow obstruction, without compensation in a terminal arterial territory. The capsular warning syndrome was first described in patients with lacunar stroke involving the internal capsule presenting repeated, stereotyped episodes of motor lacunar syndrome or sensorimotor lacunar syndrome, within 24–72 h, with complete recovery between episodes, which involved two of three body parts (face, arm, or leg), or more, without cortical symptoms [56,57]. Other studies described lacunar strokes with a similar clinical stuttering course, in other anatomical regions as the pons [58,59]. Several mechanistic interpretations of the capsular warning syndrome have been formulated, including hemodynamic failure in the presence of a stenotic perforating artery, arteriolar vasospasm, and peri-infarct depolarization [56,60]. However, the fluctuating insufficiency of a residual blood flow compensation through collateral vessels from nearby perforating arteries would also be consistent with the clinical stuttering presentation. Although perforating artery branches are not directly visible in vivo using conventional imaging, three perfusion studies, one based on CT perfusion [28] and two on MRI perfusion [45,46], provided similar results of indirect evidence of hemodynamical compensation, through retrograde blood flow filling centripetally the ischemic regions that evolved into a RSSI, suggesting the presence of microscopic collateral supply by a capillary network. In few cases, the ischemic area showed an early and anterograde filling, corresponding to normal or increased perfusion, indicating the patency of the perforating artery during image acquisition, consistent with recanalization, suggesting an embolic origin of the occlusion in a minority of patients [28].

Although small perforating vessels are not directly visible on conventional MRI, small thrombi might be spotted as blooming artifacts on gradient-echo or SWI sequences in the pathway of a perforating artery in less than 20% of patients with a RSSI [61]. However, this technique probably has low sensitivity (no gold standard is available in imaging), and specificity could be hampered by badly impaired blood–brain barrier (BBB) permeability and hemosiderin deposits. Other non-conventional techniques based on high-resolution MRI for vessel wall imaging enable the detection of non-stenotic atherosclerotic plaques occluding emerging perforating arteries, despite apparently normal vascular imaging [19,62]. The number and morphology of the perforating arteries are also evaluable using high field MRI [63]. However, the long acquisition time and sensitivity to movement artifacts limit the feasibility of these techniques in patients with acute strokes.

In the last 20 years, many authors focused their attention on vascular and endothelial dysfunction in SVD using imaging to assess vascular function measures for BBB permeability, blood flow, and vascular stiffness [4]. The endothelial dysfunction seems to have a crucial role in the pathogenesis of SVD, involving vascular inflammatory mechanisms altering the BBB permeability and extravasation of inflammatory particles into the extracellular space, causing perivascular edema and microglial dysfunction. Endothelial inflammation may increase pro-thrombotic activity, favoring microthrombosis, and affect small vessel autoregulation and capillary heterogeneity [4]. Early studies, assessing BBB permeability using dynamic contrast-enhanced MRI, showed that patients with lacunar strokes have increased BBB permeability, compared to patients with cortical strokes [64]. Other measures of vascular function as cerebrovascular reactivity appeared to be impaired in patients with SVD [65] and lacunar strokes [66] in a few cross-sectional studies. However, it is difficult to obtain these measures prior to the appearance of new subcortical infarcts in longitudinal studies, due to the relatively low rates of incident strokes and use of secondary prevention measures.

The radiological fate of RSSI is variable and hardly predictable, as one-third of RSSI evolve into a lacune, while others may disappear or leave a non-cavitated lesion [67,68]. BBB leakage into CSF appeared to be a predicting factor of cavitation in one longitudinal study [69], but further longitudinal studies might identify other risk factors. The clinical and prognostic relevance of RSSI cavitation remains to be determined, although some associations have been found with severe progression of SVD and cognitive impairment [70]. Nevertheless, most lacunes might not be related to prior clinical events and represent accidental findings on neuroimaging studies. The spatial correlation with motor and sensitive pathways is highly related to overt clinical symptoms [71], but some lacunes, even in eloquent areas, might not have been recognized clinically. Therefore, some lacunes might result from progressive injury and cavitation, which could be less clinically evident. For example, new cavities usually appear silently in the edges of white matter hyperintensities in patients with CADASIL [72], and lacunes are associated with deep medullary vein stiffening and occlusion, due to venous collagenosis [73]. 

## 4. Plasmatic Biomarkers in Patients with Lacunar Strokes

The main pathophysiological mechanisms, such as coagulation/fibrinolysis processes, endothelial dysfunction, and inflammation, involved in cerebral SVD may be assessed by measuring specific plasma biomarkers [34,38]. Patients with lacunar strokes present a pro-thrombotic state, demonstrated by overall increased plasma levels of tissue plasminogen activator (TPA), plasminogen activator inhibitor (PAI), fibrinogen, and D-dimer, compared to patients without stroke, but lower levels, compared to other stroke subtypes, characterized by larger infarct volumes [38,74]. Similarly, endothelial activation markers (homocysteine and von Willebrand factor) and leukocyte adhesion molecules (E-selectin, P-selectin, ICAM-1, and VCAM-1) expression is augmented in patients with lacunar stroke, compared to healthy controls, but not different from other stroke subtypes [38,74]. Several plasma markers of inflammation have been assessed in patients with SVD and lacunar strokes [38]. C-reactive protein (CRP) is a sensitive, but not specific, marker of systemic inflammation that has been related to stroke risk in the community-dwelling population [75] and risk of ischemic recurrence in patients who had a lacunar stroke [76,77]. Other blood markers of inflammation, such as TNF-α and IL-6, have been related to ischemic recurrence in patients with lacunar stroke [78]. However, the levels of most inflammatory markers were similar, or even lower, in patients with lacunar stroke, compared to other stroke subtypes, suggesting that most of these markers are due to the impact of the acute ischemic injury and may depend more on the stroke volume, rather than the different etiology [39]. Table 2 summarizes the potential biomarkers of lacunar strokes that have been previously studied, grouped by their primary related pathophysiological mechanism.

## 5. Insights from Translational Research

Direct evidence on SVD and small lacunar infarcts pathophysiology is particularly challenging to obtain from post-mortem studies and in neuroimaging for the low mortality and limited spatial definition, respectively. Therefore, experimental models of lacunar strokes are warranted to broaden the knowledge on the pathophysiological mechanisms and identify new therapeutic targets. However, several technical and conceptual issues may limit their translatability to humans [6,7]. First, there are relevant anatomical differences between animal models and the human brain, such as the ratio of gray/white matter and vessel distribution and density. There are different techniques to obtain small subcortical ischemic lesions, modeling lacunar infarcts, that include lesions produced by different mechanisms not consistent with SVD pathophysiology (i.e., vasoconstriction [119,120,121,122] and microembolism [123,124,125]), surgical or chemical endothelium injury leading to perforating artery occlusion [126,127], hypoperfusion produced by surgical bilateral common carotid artery occlusion [128,129], and others produced spontaneously in animals reproducing SVD model, such as hypertensive animals [6,130,131] or models of monogenic SVD (i.e., CADASIL) [132,133,134]. While the induced focal ischemic techniques are more reproducible, the spontaneous ones are difficult to trace or know the exact beginning. The main advantages and flaws of each technique are summarized in Table 3.

## 6. Prevalence and Diagnostic/Topographic Accuracy of the Lacunar Syndromes

In the 1960s, C.M. Fisher described the histopathological correlates of four clinical syndromes (pure motor, pure sensitive, ataxic-hemiparesis, and clumsy-hand dysarthria), defined as lacunar syndromes [136,137]. Some years later, J.P. Mohr et al. also described the sensorimotor syndrome corresponding to capsular-thalamic infarctions [138]. The sensorimotor syndrome represents about 20–30% of the lacunar syndromes, but it is also the one with the highest likelihood (>30%) of being caused by non-lacunar strokes (i.e., cortical strokes). The pure motor is the most prevalent lacunar syndrome (50–70%) but had a low specificity for spatial location (any point of the central motor pathway) [136,139], while the pure sensitive represents about 10–20% of lacunar syndromes [139,140] and have high specificity for ventroposterolateral thalamic infarctions. Dysarthria-clumsy hand syndrome [139,141] and ataxic hemiparesis [142,143,144] represent about 3–6% of lacunar syndromes and may correspond to RSSI, mainly in the internal capsule and pons, with few cases (<7%) corresponding to cortical strokes [145]. Other atypical syndromes have been described, including monoparesis, partial cortical, or extrapyramidal symptoms, as well as deficits due to cranial nerve nuclei involvement in brainstem infarcts [8,146]. Moreover, up to 30% of cortical strokes may also present with motor or sensitive symptoms alone mimicking subcortical infarcts. The diagnostic accuracy of the lacunar syndromes for RSSI has been addressed in several studies, most of them using MRI as the gold standard for lesion location, as detailed in Table 4. 

## 7. Neuroimaging in Patients with Suspected Lacunar Stroke

The confirmation of a RSSI in patients presenting with a lacunar syndrome requires identification on neuroimaging (either CT or MRI) of a lesion consistent with a small ischemic stroke in the territory of a single deep perforating artery (lenticulostriate, thalamoperforating, thalamogeniculate, paramedian, and deep medullary arteries), corresponding to subcortical white matter (centrum semiovale and corona radiate, internal and external capsule, and short and long fibers in the brainstem) or deep grey structures (basal ganglia, thalamus, and nuclei) in the brainstem. CT was the first technique capable of identifying small focal hypoattenuations consistent with lacunar strokes [157]. Small subcortical infarcts are hardly visible as early small ischemic changes in the first hours after symptom onset on CT scans and difficult to distinguish from older lesions in patients with SVD. The introduction of MRI for stroke assessment made possible a more precise topographic and morphologic RSSI characterization [158]. In particular, the implementation of DWI was crucial to identify recent lesions as hyperintensities (few minutes from stroke onset and remaining visible for about 3–5 weeks), while older lesions are visible in the other structural sequences [159,160]. Despite the high diagnostic accuracy of MRI, small lesions might be missed, depending on several factors, such as the magnetic field, correction of motion artifacts, and width of the consecutive slice acquisition [148]. Thus, the absence of hyperintense lesions on DWI should not rule out a lacunar stroke in the presence of a lacunar syndrome. Finally, the presence of a RSSI on MRI could represent the residual infarction of a larger perfusion deficit involving more than a perforating artery or large vessel territories, as shown in some perfusion studies [161]. In other cases, perfusion deficits in the territory of one perforating artery may be reversible [55]. A stroke results from dynamic ischemic processes, depending on several factors, such as metabolic demand, collateral blood supply, time of ischemia, and reperfusion. The snapshot provided by imaging studies might provide incomplete information that needs to be complemented by clinical assessment and appropriate complementary exams. Even so, the etiological diagnosis must be considered presumptive in lacunar stroke in the absence of a pathological confirmation and, especially, cautious in case of incomplete or inconclusive workup or when possible concomitant causes are present [162].

Identifying RSSI is particularly challenging in the acute stroke assessment for technical limitations of standard imaging techniques. However, imaging may assist clinicians in the early presumptive diagnostic orientation for assessing reperfusion in eligible patients, secondary prevention strategies, and the clinical interpretation of threatening clinical courses, such as neurological deterioration and stuttering symptoms in lacunar strokes. The most commonly adopted techniques for acute stroke assessment are based on CT imaging, while MRI is used in fewer centers and might be less practical and more time-consuming in the emergency service. Although the accuracy of non-contrast CT evaluation is extremely low in the acute phase, multimodal imaging, including CT angiography and perfusion maps, may improve the detection of small perfusion deficits corresponding to lacunar stroke. Several studies confirmed the utility of CT perfusion in lacunar stroke assessment in the past few years (Table 5), despite some important limitations, due to low sensitivity for very small infarcts and those located in the brainstem and lack of external validation and automatic detection tools. In addition, sensitivity and specificity are not comparable between studies, due to different population, selection criteria, RSSI prevalence, and study design. For example, some of the studies had extremely high specificity, probably due to pre-evaluation selection of patients or strict criteria to identify a perfusion deficit [163,164,165,166]. Nevertheless, the accuracy of non-contrast CT is highly improved by perfusion maps [43,163,164,166], which should be carefully evaluated for the presence of focal perfusion alterations, consistent with the clinical presentation.

## 8. Lacunar Stroke Management

No disease-specific treatment targeting intrinsic SVD mechanisms currently exists. Therefore, patients with lacunar stroke should receive the treatments that have demonstrated their efficacy in the acute stroke treatment and secondary prevention, such as systemic thrombolysis and antithrombotic therapies, respectively, to avoid irreversible damage in the brain. 

## 9. Intravenous Thrombolysis

Some concerns have been raised about the efficacy of this therapy in lacunar strokes, since the underlying pathophysiology might be related to mechanisms alternative to thrombosis in small vessel disease. However, the main RCTs that proved the efficacy of systemic thrombolysis in patients with stroke included patients with lacunar syndromes and showed no effect modification by the presumed vascular subtype [167]. In terms of safety, the risk of haemorrhagic complications after systemic thrombolysis in patients with lacunar infarcts is lower than in patients with other stroke etiologies; additional observational studies supported the benefit of this treatment in all stroke subtypes [168,169]. Therefore, intravenous thrombolysis is indicated for patients presenting with a lacunar syndrome in the first 4.5 h from symptom onset, indifferently of other stroke types [170]. Recent studies demonstrated that systemic thrombolysis is also effective in the extended time window (more than 4.5 h from stroke-onset) in patients with stroke viable tissue, quantified with MRI (DWI-FLAIR mismatch) and CT perfusion [171]. The MRI-based thrombolysis in the wake-up stroke trial (WAKE-UP), based on DWI/FLAIR mismatch in acute MRI evaluation, included patients with lacunar strokes, showing a benefit from IV thrombolysis, similar to large vessel strokes in a post hoc analysis [172]. Other studies (extending the time for thrombolysis in emergency neurological deficits trial, ECASS IV-ExTEND), based on penumbra quantification using CT perfusion, demonstrated the efficacy of IV thrombolysis in stroke with large volume, thus excluding patients with small subcortical infarcts [173]. Future studies should assess which perfusion maps are most accurate for selecting patients with small perfusion deficits for reperfusion in the extended time window, including those with mild symptoms and focal hypoperfusion, in the territory of a perforating artery that might be at risk of neurological deterioration [174].

## 10. Secondary Prevention

### 10.1. Antplatelets

Any single antiplatelet therapy is effective for the secondary prevention of ischemic strokes of almost all subtypes, including lacunar ischemic strokes [175]. Dual antiplatelet therapy (DAPT) has been assessed for secondary prevention of ischemic stroke, including lacunar strokes. The SPS3 trial showed that acetyl-salicylic acid (ASA) plus clopidogrel in patients with lacunar stroke in the prior six months did not reduce the incidence of new strokes but increased the risk of bleeding and death [176]. However, later trials demonstrated the efficacy of short-term DAPT started early (12–24 h) and maintained for 21–90 days, compared with single antiplatelet therapy in patients with mild strokes and TIA. The clopidogrel in high-risk patients with acute non-disabling cerebrovascular events (CHANCE) [177] and platelet-oriented inhibition in new transient ischemic attack and minor ischemic stroke (POINT) [178] trials assessed the efficacy of ASA plus clopidogrel vs. ASA alone in patients with NIHSS < 3 and <5, randomized within 24 and 12 h from stroke onset, during 21 and 90 days, respectively. More recently, the acute stroke or transient ischemic attack treated with ticagrelor or ASA for prevention of stroke and death trial (THALES) [179] assessed the efficacy of ticagrelor plus ASA in patients with NIHSS < 5, randomized within 24 h, during 30 days. In these RCTs, DAPT vs. ASA alone decreased the incidence of new strokes and cardiovascular events, despite minor increases in haemorrhagic risk, which was higher in studies with longer DAPT treatment. Nevertheless, few data are available in the subgroups of patients with lacunar strokes in these RCTs. A post hoc analysis of the CHANCE trial showed that patients with multiple strokes had lower stroke recurrences than those with a single stroke (about half of them were classified as lacunar strokes), which benefited more from DAPT vs. ASA alone [180]. Some observational studies showed that DAPT is safe in patients with lacunar infarct and early neurological deterioration, even in those who received IV tPA, and seemed to improve the functional outcomes [174,181,182]. In the SPS3 trial, patients treated with DAPT had a risk of major bleeding, compared to ASA alone, but this difference was due to extracranial hemorrhage and mainly occurred after one year of DAPT. Therefore, DAPT is not indicated in patients with a history of prior lacunar stroke. However, DAPT might be the best option for early secondary prevention in patients with mild lacunar stroke for a short period, as recommended by the American and European Guidelines [170,183]. Moreover, DAPT might limit the effect of antiplatelet resistance, measured by impedance aggregometry, to either ASA or clopidogrel in mild strokes, especially in patients with SVD [184,185]. Patients with a lacunar stroke presenting with high NIHSS might also benefit from DAPT, since they have a relatively small infarct volume, compared to cortical strokes with similar clinical severity and theoretically lower risk of haemorrhagic transformation. Imaging selection, according to stroke volume and other SVD factors that increase the risk of intracranial bleeding (i.e., the presence of multiple cerebral microbleeds on MRI), should be evaluated in the future in patients who could benefit from DAPT.

Cilostazol is a phosphodiesterase-3 inhibitor commonly used in Asiatic countries for stroke prevention with mild antiplatelet effect and other properties on endothelial dysfunction, myelin repair, neuroprotection, and inflammation. A recent meta-analysis of RCTs, comparing cilostazol vs. ASA, clopidogrel, or placebo, suggested that cilostazol is effective for stroke prevention, without increasing the hemorrhagic risk [186]. The efficacy appeared higher when treatment duration was longer than six months and in trials including more than 40% of lacunar stroke type. 

The best management of secondary prevention antithrombotic drugs in patients with lacunar stroke is still debated, and further RCTs are needed. At the moment, strong antiplatelet prevention with DAPT, instead of single antiplatelet therapy, should be considered only for mild strokes for the first few weeks after acute stroke, when the ischemic recurrence risk is higher. Future trials are warranted to confirm whether adding cilostazol to aspirin or clopidogrel for long-term prevention could offer additional protective effects, due to non-platelet activity, without increasing the hemorrhagic risk. 

Despite the efficacy of antiplatelet therapy in preventing new strokes, lacunar infarcts represent only the tip of the iceberg of SVD. The progression of the disease and appearance of related serious conditions, such as cognitive impairment, extrapyramidal syndromes, and psychiatric disorders, might require other treatments targeting alternative mechanisms in SVD.

### 10.2. Statins

The perforating arteries supplying the territory of a lacunar infarct may present arteriosclerosis with microatheromas in the proximal portions or branch atheromatous disease occluding the perforators’ origin [18] and benefit from lipid-lowering. The SPARCL trial demonstrated that, in patients with a stroke or transient ischaemic attack and LDL > 100, 80 mg of atorvastatin daily reduces the risk of new cerebrovascular events, despite a mild increase in haemorrhagic strokes. A post hoc analysis, according to stroke subtypes, showed that patients with large and small vessel occlusions benefited similarly from statin use [187], reassuring some of the concerns rose about the possible increased haemorrhagic risk in patients with SVD. However, there is no consensus about the target for LDL reduction in these patients.

### 10.3. Anti-Hypertensive Treatments

The hypertension-induced mechanisms in SVD are complex and include structural and functional maladaptation to high blood pressure and arterial pulsatility, oxidative stress, endothelial dysfunction, BBB disruption, capillary rarefaction, and impaired neurovascular coupling [188]. The effect of hypertension might not be the same in deep perforating and cortical perforating arteries. The former are more exposed to hypertensive injury, due to the high pressure gradient from the main (proximal) cerebral arteries, while the latter (deep medullary arteries penetrating the supratentorial white matter) depends on the (distal) leptomeningeal circulation, with a low pressure gradient, and might be more prone to hypoperfusion [189]. 

Avoiding hypertension is indicated in the secondary prevention of patients having a stroke [170,190]. However, the optimal blood pressure target in patients with lacunar stroke is unknown. In the SPS3 trial, intensive blood pressure treatment (systolic < 130 mmHg) in patients with lacunar infarcts was not superior to achieving standard blood pressure target (130–150 mmHg) to reduce stroke recurrence, despite a positive trend in favor of intensive blood pressure treatment [23]. Notably, the incidence of stroke recurrences in SPS3 was low in both arms, suggesting that a comprehensive management of patients with lacunar infarcts is advantageous, leaving small room for further benefit attributable to intensive blood pressure lowering. On the other hand, inducing hypotension in patients with SVD could affect the compensatory mechanism in the presence of altered arteriolar stiffness and autoregulation. The potentially harmful effect of intensive blood lowering could also be more relevant in the hyperacute phase for the potential decrease in residual compensatory perfusion flow that could accelerate the ischemic process. However, a substudy of the enhanced control of hypertension and thrombolysis stroke study (ENCHANTED) did not suggest any deleterious effect of acute blood pressure lowering, as the functional outcome in patients with lacunar stroke treated with thrombolysis was similar, independently of standard (blood pressure < 185/110 mmHg) vs. intensive (systolic target 130–140 mmHg) within one hour from randomization [191].

### 10.4. Lifestyle Interventions

Lifestyle interventions include a range of behaviorally modifiable risk factors, such as smoking, physical activity, and diet (i.e, low salt intake), that probably represent the targets with the most significant potential impact in the prevention of all subtypes of stroke [192,193]. However, few studies were conducted in the specific population of patients with a lacunar stroke. A small RCT (*n* = 71) in patients with lacunar stroke did not show differences in cardiorespiratory fitness measures in patients randomized to high-intensity interval training or moderate-intensity continuous training [194]. Nevertheless, the clear evidence from prior studies, including all subtypes of strokes, encouraging physical activity after stroke can be extrapolated for the lacunar subtype.

## 11. New Molecular Targets in Lacunar Stroke

The current management for patients presenting with a lacunar stroke is based on reperfusion treatment with thrombolytic agents and secondary antithrombotic prevention (antiplatelets), as well as other interventions on modifiable vascular risk factors (i.e., lifestyle intervention, statins, and antihypertensive drugs). However, no specific treatment for SVD exists, and new possible therapeutic targets, involving different mechanisms in SVD, are currently under investigation (Table 1).

As aforementioned, cilostazol is a promising drug in SVD for non-antiplatelet activity on BBB integrity, vasodilation, inflammation, and neuroprotection. A small RCT trial, conducted in Korea (effect of cilostazol in acute lacunar infarction, based on pulsatility index of transcranial doppler, ECLIPse), showed that adding cilostazol to aspirin in the secondary prevention of patients with lacunar stroke decreased intracranial pulsatility in the main cerebral arteries, confirming the pleiotropic effect of cilostazol beyond the antiplatelet activity [195]. The LACI-1 was a phase IIa trial that indicated the safety and tolerability of cilostazol and isosorbide mononitrate (an oxid nitric donor commonly used in angina) in patients with a lacunar stroke, also showing an improvement of vascular function in the white matter measured using cerebrovascular reactivity in blood oxygenation level dependent (BOLD)-MRI. A phase IIb trial (LACI-2) to assess the feasibility, tolerability, and provide clinical outcomes is ongoing [32].

Inflammation represents a potential target in stroke secondary prevention that could particularly fit with SVD etiology, since mechanisms other than thrombosis seem to have a pivotal role outside the acute stroke phase. The URICO-ICTUS trial showed promising results on the efficacy of a potent endogenous antioxidant molecule, such as uric acid, associated with systemic thrombolysis in acute ischemic stroke patients [41]. Although uric acid was not significantly superior to the placebo in the primary outcome measure (excellent outcome defined as modified Rankin Score 0–1 at 90-day follow-up, adjusted risk ratio 1.23 [95% CI 0.96–1.56]; *p* = 0.099), it seemed to be particularly effective in some subgroups of patients, such as women, patients with high glucose levels, and milder strokes (NIHSS < 10). However, only 5% of the patients enrolled had a lacunar stroke, and the efficacy of uric acid in this particular subgroup of stroke should be tested in future trials. Colchicine is an anti-inflammatory drug with different mechanisms of action, including microtubule disruption, neutrophile targeting, and inhibition of inflammasome-mediated IL-1β maturation [196]. The CONVINCE is a phase III PROBE design trial that will assess the efficacy of colchicine in patients with non-severe, non-cardioembolic stroke or high-risk TIA [40]. Canakinumab is a therapeutic monoclonal antibody targeting interleukin-1β innate immunity that reduced cardiovascular events in patients with myocardial infarction and high CRP levels. However, the efficacy in the stroke population has not been assessed yet [42]. 

## 12. Future Directions

Systemic thrombolysis in patients with lacunar stroke attending later than 4.5 h from symptom onset is not a marginal issue. It often occurs in patients with stuttering and progressively worsening symptoms. However, the thrombolysis indication in this subgroup of patients is still uncertain, despite the encouraging results from the WAKE-UP trial [172]. Perfusion studies in the acute stage could also identify patients with mild non-disabling symptoms but an evident, relatively large perfusion deficit in the territory of the perforating artery, which may present neurological deterioration and possibly benefit from thrombolysis. While MRI is not feasible in the acute stroke assessment in most stroke centers, automatic CT perfusion software packages are not helpful for detecting small perfusion deficits, and visual assessment could be challenging, especially for untrained readers. Therefore, multicentric studies, pooling large series of patients with lacunar strokes, are warranted to improve the automatic detection of small perfusion alterations and validate the results obtained in smaller, single-center cohorts.

Secondary prevention measures, mainly based on antiplatelet and vascular risk factors control, are not much different from other non-cardioembolic ischemic strokes. However, long-term DAPT is hazardous in patients with SVD for increasing the bleeding risk, while the risk of late stroke recurrences and SVD progression is higher in patients with lacunar strokes than in those with large vessel stroke. For these reasons, the research of new drugs targeting non-thrombotic mechanisms of SVD is particularly encouraging for the long-term, secondary prevention of lacunar stroke. 

## 13. Limitations of the Review

This review is to be considered a broad overview on several pathophysiological, clinical, diagnostic, and therapeutic issues concerning lacunar stoke. Therefore, the main limitation of the review is the lack of a systematic literature search methodology. However, the main aim of the review is to provide a personal view for clinicians and highlight opportunities for future lines of research, including systematic reviews and meta-analyses on specific topics, such as biomarkers clinical relevance and secondary prevention approaches.

## 14. Conclusions

Lacunar ischemic strokes are not only a prevalent type of stroke but could also be the first clinical manifestation of SVD, which causes severe physical and cognitive impairment in the long-term. However, not all small subcortical lesions are due to SVD, and improvement in the diagnostic work-up, including advanced imaging, is desirable to exclude other concomitant causes. Translational research is often necessary to develop of new treatments, but optimal animal models of lacunar strokes, mimicking the same underlying mechanisms, are lacking at the moment. On the clinical side, surrogate markers of vascular functions in SVD in neuroimaging are not fully developed and validated. More efforts are needed in the future to encourage the external validation of new SVD markers and include them in large multicenter prospective studies, including RCTs. Using homogenous definitions of SVD markers, standards in the acquisition and processing of SVD imaging are crucial for interpretating and comparing different studies.

## Figures and Tables

**Table 1:** Possible mechanisms involved in lacunar stroke pathogenesis.

Mechanism	Description	Evidence	Unsolved Issues	Possible Intervention Target
Hypertensive arteriosclerosis	Progressive hypertensive-related arteriosclerotic injury. Superposed microthrombosis may lead to complete arteriolar occlusion.	Typical histopathological findings in perforating arteries. Indirect evidence from high field MRI techniques [19,20].	Non hypertensive patients may also present with lacunar stroke [21]. In vivo radiological confirmation of small artery wall alterations are not available.	Hypertension is the most modifiable risk factor for stroke secondary prevention [22]. In patients with lacunar strokes, intensive vs. standard blood pressure reduction did not reduce the risk of all stroke recurrency, although it reduced the risk of intracranial hemorrhage (SPS3) [23].
Atherosclerosis (branch atheromatous disease)	Atherosclerotic plaques in the main cerebral vessel may occlude the orifice of perforating arterioles [24,25].	Anatomopathological studies [18]. Small plaques are also visible using high field MRI techniques for vessel wall assessment [24].	Atherosclerosis in large vessel arteries may represent an epiphenomenon.	Lipid lowering is effective for reducing stroke recurrence in non-cardioembolic strokes (SPARCL trial) [26]. Other new drugs aimed to stabilize the inflammatory process in atherosclerosis, which might represent a promising therapeutic target.
Microembolisms	Small emboli, either from proximal atherosclerotic plaques or cardiac source, may produce single or multiple small subcortical infarcts.	Perforating arteries in lacunar strokes may be patent in pathology studies [8] and advanced 7T MRI techniques [27]. Increased blood flow on CT perfusion suggests recanalization of an embolic occlusion of a perforating artery [28]. Subcortical infarcts in animal models produced by microembolism [6].	There is an association between atrial fibrillation, load of subcortical infarcts, and WMH [29], but direct evidence of embolism is lacking. Multiple RSSIs do not exclude mechanisms related to SVD (about 20% of RSSI present multiple infarcts, especially in patients with severe SVD [30]).	Treatments aimed to stabilize active plaques or anticoagulant treatment, in case of mayor embolic source. Prothrombotic state (i.e., acute cancer), marantic, or infectious endocarditis should be ruled out in patients with multiple subcortical strokes.
Chronic global cerebral hypoperfusion	Chronic hypoperfusion of distal vascular territories may lead to progressive ischemia in the white matter. Small infarctions may occur in the edges of WMH and contribute to SVD progression.	In animal models, small subcortical infarcts may be produced by bilateral carotid occlusions [6].	The causal relationship between hypoperfusion and SVD progression in longitudinal studies is controversial [31], as hypoperfusion might also be also secondary to reduced metabolism in WMH.	Vasodilatory drugs to increase brain perfusion: mononitrate isosorbide, nitric oxide. (LACI-2) [32].
Inflammation, endothelial dysfunction, and BBB disruption	Endothelial dysfunction may trigger the pro-inflammatory mechanisms promoting pro-thrombotic agents, microglial activation, altered neurovascular homeostasis, and impaired coupling between metabolic demand and nutrient supply.	Markers of BBB leakage in pathology studies [33,34]. Association between the number of lacunes and inflammatory blood markers [35]. BBB permeability on dynamic contrast enhanced MRI is increased in lacunar strokes, compared to cortical strokes [36].	Some studies on post-mortem brain samples did not confirm the association of markers of endothelial dysfunction or BBB leakage and SVD [37,38]. A causal relationship with focal BBB leakage prior lacunar strokes is to be determined. BBB permeability variations are mild and difficult to measure in SVD. Blood markers of endothelial dysfunction and inflammation are are not specific of lacunar stroke subtype [39].	Anti-inflammatory drugs: colchicine in non-cardioembolic strokes (CONVINCE) [40], uric acid (URICO-ICTUS) [41], and canakinumab [42].
Focal hypoperfusion and compensatory blood flow in acute perforating artery occlusion	Abrupt reduction in blood flow after perforating artery occlusion, regardless the causing mechanisms (either intrinsic SVD or atheroembolic). The extent and the time to establish infarction may depend on factors such as compensatory blood flow through capillary network and cerebrovascular reserve.	Perfusion studies show persistence of residual blood flow, in the territory of perforating arteries corresponding to RSSI [43,44]. Sequential imaging from row perfusion sequences may show retrograde flow, suggesting collateral circulation involvement in RSSI [28,45,46] Microscopic studies showed a dense capillary network, linking contiguous perforating arteries and few arteriolar anastomoses [47].	Lack of direct evidence of perforating artery occlusion and recruiting collateral circulation in RSSI	Thrombolysis in lacunar stroke would not be effective without compensatory mechanisms maintaining the tissue viable until recanalization. Perfusion imaging-based thrombolysis, outside of the conventional time window, may also be effective in patients with RSSI. Vasodilatory agents may improve collateral recruitment. Neuroprotective agents may reach the ischemic area through retrograde in the territory supplied by an occluded perforating artery.

BBB: blood–brain barrier; CONVINCE: colchicine for prevention of vascular inflammation in non-cardioembolic stroke; LACI-2: lacunar intervention trial-2; RSSI: recent small subcortical infarcts; SVD: small vessel disease; SPARCL: stroke prevention by aggressive reduction in cholesterol levels; SPS3: secondary prevention of small subcortical stroke trial; URICO-ICTUS: uric acid in patients with acute stroke trial; WMH: white matter hyperintensities.

**Table 2:** Plasma biomarkers in lacunar stroke.

Mechanism	Molecule	Findings	References
Coagulation and fibrinolysis	Tissue plasminogen activator (TPA)	- Higher in lacunar stroke vs. non-stroke, acutely and chronically - Similar in lacunar stroke vs. non-lacunar stroke, acutely and chronically	Lindgren, 1996 [79] Salobir, 2003 [80] Jood, 2005 [81]
	Plasminogen activator inhibitor (PAI)	- Higher in lacunar stroke vs. non-stroke, acutely and chronically - Similar in lacunar stroke vs. non-lacunar stroke, acutely and chronically	Lindgren, 1996 [79] Salobir, 2002 [80] Jood, 2005 [81] Yokokawa, 2008 [82] Ilhan, 2010 [83]
	Fibrinogen	- Similar in lacunar stroke vs. non-stroke, acutely - Higher in lacunar stroke vs. non-stroke, chronically - Lower in lacunar stroke vs. non-lacunar stroke, acutely and chronically	Kilpatrick, 1993 [84] Beamer, 1995 [85] Bath, 1998 [86] Kataoka, 2000 [87] Salobir, 2003 [80] Jood, 2008 [81] Álvarez-Pérez, 2011 [88] Beer, 2011 [89] Zhang, 2011 [90] Datta, 2014 [91]
	D-dimer	- Higher in lacunar stroke vs. non-stroke, acutely and chronically - Lower in lacunar stroke vs. non-lacunar stroke, acutely and chronically	Takano, 1992 [92] Kataoka, 2000 [87] Ajeno, 2002 [93] Salobir, 2003 [80] Ilhan, 2010 [83] Montaner, 2008 [94] Brouns, 2009 [95] Isenegger, 2010 [96] Álvarez-Pérez, 2011 [88]
Endothelial dysfunction	Homocysteine	- Higher in lacunar stroke vs. non-stroke, acutely and chronically - Similar in lacunar stroke vs. non-lacunar stroke, acutely	Eikelboom, 2000 [97] Hassan, 2004 [98] Parnetti, 2004 [99] Khan, 2007 [100] Khan, 2008 [101] Yokokawa, 2008 [82] Beer, 2011 [89] Jeong, 2011 [102] Pavlovic, 2011 [103] Lavallée, 2013 [104]
	Von Willebrand factor (vWF)	- Higher in lacunar stroke vs. non-stroke, acutely - Lower in lacunar stroke vs. non-lacunar stroke, acutely	Beer, 2011 [89] Hanson, 2011 [105] Lavallée, 2013 [104]
	E-selectin	- Higher in lacunar stroke vs. non-stroke, acutely Similar in lacunar stroke vs. non-lacunar stroke - Similar in lacunar stroke vs. non-lacunar stroke	Kozuka, 2002 [106] Beer, 2011 [89]
	P-selectin	- Higher in lacunar stroke vs. non-stroke but only in some studies - Similar in lacunar stroke vs. non-lacunar stroke	Bath, 1998 [86] Kozuka, 2002 [106] Tsai, 2009 [107] Ilhan, 2010 [83] Turgut, 2011 [108] Lavallée, 2013 [104]
	Intercellular adhesion molecule 1 (ICAM-1)	- Higher in lacunar stroke vs. non-stroke, acute and chronically - Similar in lacunar stroke vs. non-lacunar stroke, acutely	Castellanos, 2002 [109] Hassan, 2003 [98] Supanc, 2011 [110] Rouhl, 2012 [111]
	Vascular cellular adhesion molecule 1 (VCAM-1)	- Similar in lacunar stroke vs. non-lacunar stroke, acutely	Supanc, 2011 [110] Rouhl, 2012 [111] Brwon, 2015 [112]
Inflammation	C-reactive protein (CRP)	- Higher in lacunar stroke vs. non-stroke, acutely and chronically - Similar in lacunar stroke vs. non-lacunar stroke, acutely and chronically	Ladenvall, 2006 [81] Yokokawa, 2008 [82] Montaner, 2008 [94] Nakase, 2008 [113] Álvarez-Pérez, 2011 [88] Beer, 2011 [89] Turgut, 2011 [108] Mitaki, 2016 [114]
	Tumor necrosis factor α (TNFα)	- Higher in lacunar stroke vs. non-stroke, acutely	Castellanos, 2002 [109] Domac, 2007 [115] Boehme, 2016 [78]
	Interleukin 6 (IL-6)	- Higher in lacunar stroke vs. non-stroke, acutely - Lower in lacunar stroke vs. non-lacunar stroke, acutely	Beamer, 1995 [85] Vila, 2000 [116] Castellanos, 2002 [109] Salobir, 2004 [117] Domac, 2007 [115] Guldiken, 2008 [118] Boehme, 2016 [78]

**Table 3:** Experimental models of stroke modeling lacunar infarcts.

Mechanism	Techniques	Description	Advantages	Disadvantages	References
Vasoconstriction of perforating arteries	Endothelin-1, nitric oxide synthase inhibitor, and L-NAME	Strong vasoconstrictive action that affects several microvessels	Small subcortical infarcts	Multiple vessels affected at once	Horie, 2008 [119] Capone, 2011 [120] Cipolla, 2013 [135] Cui, 2013 [122]
Embolism	Microspheres, microthrombi injection, atheroemboli, black beads, preformed clots, and silicone rubber cylinders	Injection of different materials in the carotid to produce micro-occlusions by lodging in brain vessels	Multiple subcortical infarcts	Mostly cortical infarcts, mechanism not related to SVD	Rapp, 2003 [123] Wang, 2012 [124] Silasi, 2015 [125]
Spontaneous lesions	High salt, spontaneous in SHRSP, surgical narrowing of the aorta, and genetic mutations in the renin-angiotensin system	Mice breeds with an increased risk of stroke, genetically or surgically induced	Mechanism consistent with hypertensive SVD	Difficult to track lesion location and timing	Hainsworth, 2008 [130] Bailey, 2009 [6] Mencl, 2013 [131]
Perforating artery occlusion	Surgery (pial vessel disruption model), sodium laurate thrombosis, and photothrombosis	Endothelium damage and thrombosis, using toxic substances or surgical models	Accurate localization of the lesions	Strokes larger than lacunar infarcts	Walz, 2017 [126] Wen, 2019 [127]
Transient large vessel occlusion	Bilateral common carotid artery occlusion	Repeated transient large vessel occlusion, followed by reperfusion	Mechanism reflects the hypoperfusion in SVD	Lesions are not related to small vessel pathology	Choi, 2018 [128] Kwon, 2015 [129]
Genetic models	CADASIL mouse models, COL4A1/2 mouse models	Studies in mice with rare genetic disorders which make them prone to SVD	Mechanism related to genetic SVD etiology	Not enough brain affection	Ayata, 2010 [132] Joutel, 2011 [133] Ruchoux, 2003 [134]

CADASIL: cerebral autosomal dominant arteriopathy with subcortical infarcts and leukoencephalopathy; COL4A1/2: collagen type 4 alpha 1 chain gene; L-NAME: N-nitro-L-arginine methylester; SHRSP: spontaneously hypertensive stroke-prone rats; SVD: small vessel disease.

**Table 4:** Lacunar syndrome: correspondence with imaging findings.

Reference	Cohort	Lacunar Syndrome, *n* (%)	Cortical Syndrome, *n* (%)	RSSI/NonLacunar Syndrome, *n* (%)	Non-RSSI/Lacunar Syndrome, *n* (%)	Lacunar Syndrome/DWI Negative, *n* (%)	Cortical Syndrome/DWI Negative, (%)	Lacunar Syndrome Positive Predictive Value
Potter, 2010 [147]	313	79 (25)	136 (43)	21/93 (23)	7/44 (16)	35/79 (44)	43/136 (32)	46% ° 84% °°
Ay, 1999 [148]	62	62 (100)	-	-	10/62 (16)	9/62 (14)	-	68%
Stapf, 2000 [149]	54	54 (100)	-	-	3/54 (6)	0/54 (0)	-	94%
Lindgren, 2000 [150]	23	23 (100)	-	-	2/23 (8)	1/23 (4)	-	86%
Seifert, 2005 [151]	93	41 (44)	POCS (39) PACS (15) TACS (1)	15/93 (16)	-	-	-	44% ^ 83% ^^
Wessels, 2005 [152]	73	73 (100)	-	-	30/73 (38)	0/73 (0)	-	58%
Arboix, 2010 * [153]	879	879 (100)	-	-	146/879 (17)	-	-	83%
Altmann, 2014 [154]	119	119 (100)	-	-	16/119 (13)	17/86 (20)	-	60% · 65% ··
Giacomozzi, 2019 [155]	1796	478 (26)	1313 (74)	346/1313 (26)	104/478 (21)	-	-	78%
Arba, 2020 ** [156]	568	330 (58)	238 (42)	59/238 (25)	102/330 (31)	-	-	25% *** 75% ****

* Diagnostic imaging was MRI (41%) and CT (59%); ** CT scan as follow-up imaging; *** worst-case scenario (negative CT scan considered as non-RSSI); **** best-case scenario (negative CT scan considered as RSSI). Adding NIHSS < 7 to lacunar syndrome improved the positive predictive value to 97% for both worst- and best-case scenarios. ° Worst-case scenario (negative DWI considered as non-RSSI). °° Values only considering DWI-confirmed stroke. ^ Values including only LACS. ^^ Values including both LACS and POCS. · Values including both MRI and CT follow-up imaging. ·· Values including only MRI follow-up imaging. LACS: lacunar syndrome; POCS: posterior occlusion circulation syndrome; TACS: total anterior circulation syndrome. Modified from Potter et al., 2010 [147].

**Table 5:** Studies assessing the diagnostic accuracy of CT perfusion in patients with lacunar stroke or confirmed RSSI.

Reference	Population	Perfusion Maps	Main Findings
Rudilosso, 2015 [43]	A total of 33 patients with lacunar syndrome (16 lacunar strokes, 13 non-lacunar strokes, and 4 no ischemic lesions). Lacunar stroke defined as infarct volume <1.767 cm^3^ (the volume of a sphere with a diameter 1.5 cm) on DWI.	Postprocessing software: CT Neuro Perfusion Syngo.via (Siemens Healthcare GmbH) for visual assessment. MIStar (Apollo Medical Imaging Technology, Melbourne, Australia) for core/penumbra threshold analysis. Perfusion maps: CBF, CBV, MTT, Tmax, TTP, TTD, and MIP.	SE and PPV for lacunar stroke higher than non-contrast CT (63% vs. 19%). CTP was more sensitive for supratentorial lesions, compared with infratentorial lesions (65% versus 16%). SP was low (20%) and influenced by low lacunar stroke prevalence. TTD was the most informative map for the identification of ischemic lesions.
Das, 2015 [163]	A total of 88 patients with lacunar syndrome (after excluding stroke mimics). RSSI: 59/88 (67%).	Postprocessing software: GE Healthcare Perfusion maps: CBV, CBF, and MTT.	SE56%, SP 83%. CTP increased the diagnostic performance 5-fold over non-contrast CT. MTT were the most informative maps to identify RSSI.
Benson, 2016 [164]	A total of 113 patients: 37 with ischemic lesions on DWI < 20 mm in maximum diameter (either cortical or subcortical) and 76 without ischemic lesions. Ischemic lesions > 20 mm, and patients treated with iv tPA were excluded from the analysis.	Postprocessing software: Vitrea workstation (Vital Imaged, Minnetonka, Minnesota) Perfusion maps: TTP, MTT, CBV, and CBF.	TTP were the maps with highest SE (49%), and lowest for non-contrast CT (3%). SP was high regardless the map evaluated (all >97%). The perfusion lesions on CTP appeared larger than the lesion on DWI.
Tan, 2016 [161]	A total of 182 patients with ischemic strokes (31 single subcortical, 9 multiple subcortical, 34 cortical only, 33 non-confluent cortical-subcortical, and 75 confluent cortical-subcortical).	Postprocessing software: Advantage Windows (GE Medical Systems) and Extended Brilliance Workspace (Philips Healthcare, Best, Netherlands) Perfusion maps: MTT.	39% of the RSSI (single subcortical) on DWI had a perfusion deficit. However, for 67% of them, the perfusion deficit was larger than the DWI lesion and were associated with a large vessel occlusion on CT angiography.
Cao, 2016 [165]	A total of 62 patients: 32 with RSSI and 30 without lesions on DWI.	Postprocessing software: RAPID iSchemicView (Menlo Park, CA, US) Perfusion maps: CBF, CBV, MTT, and Tmax.	MTT showed 56% SE. No false positive perfusion images were rated.
García-Esperón, 2021 [166]	A total of 106 patients with lacunar syndrome: RSSI, 33 cortical and 14 posterior fossa strokes. Patients without lesions on DWI were excluded.	Postprocessing software: MIStar (Apollo Medical Imaging Technology, Melbourne, Australia) Perfusion maps: CBF, CBV, MTT, and DT.	42% SE, 80% SP for RSSI. Visual inspection of CTP maps had higher SE than the automated method (42% vs. 6%). Sensitivity on non-contrast CT was very low (<4%).

CBF: cerebral blood flow; CBV: cerebral blood volume; CTP: CT perfusion; DT: delay time; DWI: diffusion-weighted imaging; MIP: maximum intensity projection; MTT: mean transient time; PPV: positive predictive value; RSSI: recent small subcortical infarct; SE: sensitivity; SP: specificity; Tmax: time to maximum; TTD: time to drain; TTP: time to peak.
